# Real-World Safety Profile of Biologic Drugs for Severe Uncontrolled Asthma: A Descriptive Analysis from the Spanish Pharmacovigilance Database

**DOI:** 10.3390/jcm13144192

**Published:** 2024-07-18

**Authors:** Carlos Boada-Fernández-del-Campo, Marcelino García-Sánchez-Colomer, Eduardo Fernández-Quintana, Paloma Poza-Guedes, Jaime Leonardo Rolingson-Landaeta, Inmaculada Sánchez-Machín, Ruperto González-Pérez

**Affiliations:** 1 Autonomous Pharmacovigilance Center of the Canary Islands (CAFV), Hospital Universitario de Canarias, 38320 Santa Cruz de Tenerife, Spain; boada.carlos@gmail.com (C.B.-F.-d.-C.); mgarciasanchez@gsccanarias.com (M.G.-S.-C.); efernandez@gsccanarias.com (E.F.-Q.); 2 Canary Islands Health Service, Spanish Pharmacovigilance System for Medicines for Human Use (SEFV-H), 38200 Santa Cruz de Tenerife, Spain; 3 Clinical Pharmacology Service, Hospital Universitario de Canarias, 38320 Santa Cruz de Tenerife, Spain; leonardorolingson@gmail.com; 4 Allergy Department, Hospital Universitario de Canarias, 38320 Santa Cruz de Tenerife, Spain; pozagdes@hotmail.com (P.P.-G.); zerupean67@gmail.com (I.S.-M.); 5 Severe Asthma Unit, Hospital Universitario de Canarias, 38320 Santa Cruz de Tenerife, Spain; 6 Instituto de Investigación Sanitaria de Canarias (IISC), 38320 Santa Cruz de Tenerife, Spain; 7 Immunotherapy Unit, Hospital Universitario de Canarias, 38320 Santa Cruz de Tenerife, Spain

**Keywords:** severe asthma, biologic therapy, safety, adverse event, pharmacovigilance

## Abstract

**Background**: The present investigation provides a thorough analysis of adverse drug reactions (ADRs) reported in the Database of the Spanish Pharmacovigilance System (FEDRA) for biologic medications primarily indicated for severe refractory asthma, including omalizumab, mepolizumab, reslizumab, benralizumab, dupilumab, and tezepelumab. Our main objective was to identify ADRs not documented in the drugs’ Technical Sheets (summary of product characteristics, SmPC), potentially indicating unrecognized risks meriting pharmacovigilance attention. **Methods**: Data spanning from each drug’s market introduction until 22 January 2024, were analyzed, sourced from direct submissions to the Spanish Pharmacovigilance System, industry communications, and literature reviews. We evaluated notifications impartially to ensure a comprehensive review of all the ADRs associated with these medications. **Results**: This investigation underlines the critical role of post-marketing surveillance in enhancing patient safety. It emphasizes the necessity for healthcare professionals to report ADRs comprehensively to foster a robust pharmacovigilance system. Furthermore, the study highlights gaps between the reported ADRs and the information provided in SmPCs, signaling potential areas for improvement in drug safety monitoring and regulatory oversight. **Conclusions**: Finally, these findings may contribute to informed decision making in clinical practice and regulatory policy, ultimately advancing patient care and safety in the management of severe uncontrolled asthma.

## 1. Introduction

### 1.1. Background

Asthma, a significant non-transmissible respiratory condition, affects 1–18% of the world population [1,2] and stands as the most prevalent chronic disease among children globally. Despite advancements in asthma management, 3.5% to 5% of individuals with asthma prove challenging to treat, leading to increased medication usage, frequent emergency department visits, and hospitalizations, representing >50% of the direct total asthma cost [3,4,5]. Current guidelines indicate that subjects with the severe forms of the disease require treatment with high-dose inhaled corticosteroids (ICSs) along with a second controller medication—and/or systemic corticosteroids—to prevent asthma from becoming uncontrolled or remaining uncontrolled despite strictly adhering to therapy [6]. This approach reflects a departure from the traditional model of asthma as a singular entity towards a more intricate biological network where severe asthma (SA) is categorized based on observable features resulting from complex interactions between hereditary, environmental, and behavioral influences. Moreover, the enhanced comprehension of SA immunopathogenesis has progressed to the characterization of distinct endotypes classified as either type 2 (T2)-high or T2-low according to the varying levels of expression of specific cytokines like IL-4, IL-5, and IL-13 [7]. Clinically, T2-high asthma has been categorized into three distinct phenotypes, comprising early-onset allergic asthma, late-onset eosinophilic asthma, and nonsteroidal anti-inflammatory drugs (NSAIDs)-exacerbated respiratory disease (NERD) [8,9,10,11,12]. In contrast, T2 Low asthma, has been clinically grouped based on factors such as exposure to smoking, obesity, and/or age, being immunologically distinguished by the activation of inflammatory pathways involving T helper-lymphocytes type 1 (Th1) and Th17 cells as well as cytokines like IL-6, IL-8, IL-17, and IL-22 along with epithelial-derived cytokines [13,14,15,16]. Despite T2 inflammation being preponderant in SA, the assignment to a specific asthma phenotype may not remain consistent over time because there is often substantial overlap (>70%) observed among different asthma traits [17,18,19,20]. The advantage of classifying severe asthma, first discussed in 2008, lies in the possibility of targeting each endotype with specifically tailored therapy to address the underlying pathogenic molecular mechanisms [17,21].

### 1.2. Overview of Biological Agents for Asthma

In recent decades, the advent of biologics, defined by the European Medicines Agency (EMA) as a medicine whose active substance is made by a living organism [22], has revolutionized the management of SA, particularly in those individuals with the T2 endotype [23,24,25]. This transformative shift has altered our expectations for SA, enabling the exploration of the potential to alter the disease trajectory and even induce remission [26,27].

In Spain, the available biologics—formerly approved by the EMA—for severe uncontrolled asthma include six monoclonal antibodies, which are briefly described as follows:

Omalizumab (Xolair^®^) is a recombinant monoclonal antibody specifically designed to target free IgE, preventing its interaction with the high-affinity FcεRI receptors found on the surface of basophils and mast cells [28,29]. Omalizumab treatment should be considered only for patients 6 years of age and older with asthma convincingly mediated by IgE—i.e., a positive skin test or in vitro reactivity to a perennial aeroallergen—and whose symptoms are inadequately controlled with inhaled corticosteroids [30].

Mepolizumab (Nucala^®^) is a humanized IgG1 monoclonal antibody that directly binds to IL-5, preventing its interaction with the alpha chain of the IL-5 receptor from eosinophils and basophils [31,32]. First authorized in Spain in 2015, it is currently indicated as an additional treatment for severe refractory eosinophilic asthma in patients 6 years of age and older [33].

Reslizumab (Cinqaero^®^), the second available anti-IL5 biological agent in 2016, is indicated as an additional treatment in adult patients with severe eosinophilic asthma inadequately controlled with high-dose inhaled corticosteroids plus another maintenance treatment medication [34]. Reslizumab is solely designated for hospital use due to its requirement for intravenous administration.

Benralizumab (Fasenra^®^) is a humanized and afucosylated monoclonal antibody that binds with high affinity and specificity to the alpha subunit of the receptors for IL-5 specifically expressed on the surface of eosinophils and basophils [35]. Authorized in Spain in 2018, it serves as an additional treatment in adult patients with severe eosinophilic asthma who are not adequately controlled despite high-dose corticosteroid treatment and long-acting beta-2 agonists [36].

Tezepelumab (Tezspire^®^) is a human monoclonal antibody produced in Chinese hamster ovary (CHO) cells using recombinant DNA technology that blocks circulating thymic stromal lymphopoietin (TSLP) and prevents receptor binding. Authorized in Spain in 2022, tezepelumab is indicated as an additional maintenance treatment in adults and adolescents aged 12 years and older with severe asthma who are not adequately controlled despite high-dose inhaled corticosteroids in combination with another maintenance treatment [37].

Dupilumab (Dupixent^®^) is a recombinant monoclonal antibody that inhibits the signaling of interleukin-4 (IL-4) and interleukin-13 (IL-13) which are involved in type-2 inflammation. Authorized in Spain in 2017, it is indicated as an additional maintenance treatment in adults and adolescents aged 12 years and older with severe asthma characterized by type-2 inflammation, evidenced by elevated blood eosinophils and/or elevated FeNO, who are not controlled with high-dose inhaled corticosteroids in combination with another maintenance treatment [38].

### 1.3. Post-Approval Pharmacovigilance of Biological Drug in Asthma

While these agents are deemed safe upon authorization by the EMA, it should be noted that the use of any drug can lead to an increase in the frequency of reported adverse drug reactions (ADRs) and may reveal previously unknown risks, emphasizing the need for continued surveillance, especially given the lack of long-term safety data [39,40]. The Spanish System of Human Use Medicines Pharmacovigilance (SEFV-H, for its acronym in Spanish), the organization responsible for pharmacovigilance activities in Spain, gathers data on notifications (or reports) of suspected ADRs to medicines, registering them all in a database named Farmacovigilancia Española, Datos de Reacciones Adversas (FEDRA, for its acronym in Spanish). This information leads to the generation of so-called signals, which represent an association between a drug and an ADR previously unknown but considered plausible based on quantitative and qualitative data, judged to have sufficient likelihood to warrant further verification action [41]. Pharmacovigilance signals allow the generation of new hypotheses about medication safety post-marketing [42,43,44], underscoring the essential importance of healthcare professionals’ participation in spontaneous reporting programs for suspected ADRs.

### 1.4. Justification and Aim of the Study

The Allergy Department at the Canary Islands University Hospital, Tenerife, Spain, identified three cases of severe arthralgia related to the use of mepolizumab and reported them to the Autonomous Pharmacovigilance Center (CAFV, for its acronym in Spanish). In February 2023, the Canary Islands CAFV presented a signal titled “Mepolizumab and arthralgia” to the Technical Committee of SEFV-H, as this association was not reflected in the Nucala^®^ Summary of Product Characteristics (SmPCs), and arthralgia was considered as a new risk. This signal was communicated to the Pharmacovigilance Risk Assessment Committee (PRAC) of the EMA, prompting a request for all available information from the pharmaceutical laboratory that markets mepolizumab for evaluation in the next Periodic Safety Report (PSR).

Consequently, it was considered of interest to know the profile of the notifications of suspected ADRs to biological medications specifically designated for SA. Thus, the objective of this study is to examine all the reports of suspected ADRs related to any of the biological medications approved for SA in Spain, providing a complete summary of the most significant ADRs that are not currently included in their SmPC, and which, due to their severity, are likely to generate a pharmacovigilance signal.

## 2. Materials and Methods

### 2.1. Biological Medicines under Study

It is important to note that while severe refractory asthma is the primary indication for the investigated drugs—namely omalizumab, mepolizumab, reslizumab, benralizumab, dupilumab and tezepelumab—the ADRs included in this analysis also correspond to other currently approved indications for these biological drugs in Spain. This approach facilitates a comprehensive investigation of the complete profile of ADRs associated with their current clinical use (Table 1).

### 2.2. Data Source and Analysis of Cases Registered in the FEDRA Database

This investigation conducts a comprehensive retrospective observational analysis of the ADRs documented in FEDRA, spanning data from its origin in 1983 to 22 January 2024. FEDRA aggregates over 590,000 individual Case Safety Reports (ICSRs) of suspected ADRs submitted by healthcare professionals, citizens, and Marketing Authorization Holders (MAHs), including cases identified through the EMA’s Medical Literature Monitoring (MLM) service. Methodologically, the study employed a dual approach for analysis. Qualitatively, each case underwent meticulous review to assess associations between the reported ADRs and biologics primarily indicated for severe refractory asthma. It is essential to mention that each case in the study may originate from various sources, including patients and healthcare professionals like pharmacists; primary or specialty care physicians; and nurses. Additionally, individual cases often involve multiple medications administered to the same patient, leading to several reported ADRs or symptoms arising from these reactions. As a result, the total number of cases does not directly correspond to the number of unique ADRs, nor does the count of medications necessarily match the reported cases. In this regard, the statistical findings should be interpreted as the “degree of imputability” of the case series, determining whether the observed associations merit further investigation. This assessment is followed by an epidemiological evaluation that considers factors such as the temporal relationship between drug administration and adverse events, symptom improvement upon the discontinuation of the medication, the exclusion of alternative causes, and the identification of confounding factors and biases. Positive re-exposure to the drug in a case would strengthen the imputability of the reported signal [43,44].

Quantitatively, disproportionality metrics such as the Reporting Odds Ratio (ROR), Information Component (IC), and Chi-Square (X2) were calculated to assess whether the observed ADRs occurred more frequently than expected, relative to the overall reporting in the database [42,45]. Signal evaluation criteria included assessing temporal relationships between drug administration and ADR onset, observing symptom improvement upon drug cessation, excluding alternative causes, and noting the instances of positive re-exposure.

The interpretation of the findings adhered to guidelines set forth by the SEFV-H, emphasizing the cautious interpretation of ADR notifications and the role of pharmacovigilance in signaling potential risks rather than confirming causal relationships or calculating incidence rates [46]

### 2.3. Medical Dictionary for Regulatory Activities (MedDRA)

**Figure 1:**
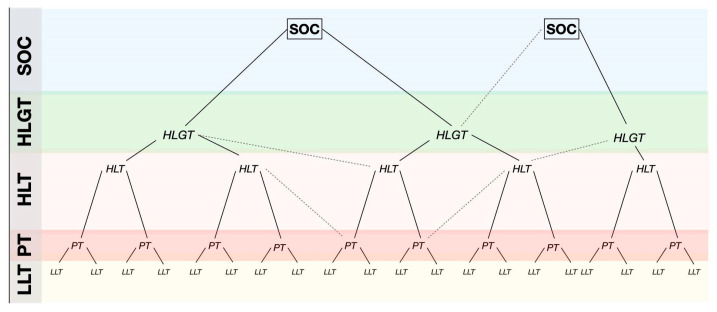
The multiaxial structure of MedDRA. Abbreviations: SOC: System Organ Classes. HLGT: High-Level Group Term. HLT: High-Level Term. PT: Preferred Term. LLT: Low-Level Term.

FEDRA utilizes the MedDRA terminological dictionary (Medical Dictionary for Regulatory Activities) for the coding of reported cases. This dictionary organizes reaction terms into a hierarchical structure consisting of five levels: the highest hierarchical level being the System Organ (SOC), encompassing 28 categories, while the lowest level comprises clinical symptoms known as Lowest Level Term (LLT), totaling 80,000. Preferred Terms (PTs) sit one level above and are initially analyzed. Higher than PT are High-Level Term (HLT) and High-Level Group Term (HLGT), which categorize terms based on different clinical characteristics. It should be noted that a term may be included in multiple hierarchical groupings, known as a “multiaxial structure” (Figure 1).

Additionally, the dictionary provides Standardized MedDRA Queries (SMQs) for specific clinical and analytical purposes that can be expressly defined for specific scenarios. Therefore, the results of this study are presented not only based on MedDRA´s hierarchical structure but also on the combination of terms most relevant from a clinical perspective.

### 2.4. Institutional Review Board Statement

This article is derived from the reviews of the existing databases, guidelines, and literature, and does not involve any studies regarding human participants or animals. These data are anonymized data, and their use has been carried out in accordance with the procedures of the Spanish Pharmacovigilance System and the Guide for the use of FEDRA data by the Autonomic Centers of the SEFV-H [47].

## 3. Results

### 3.1. Global Findings from the Analysis of Overall Data

Suspected ADRs reported in FEDRA up to 22 January 2024, attributed to drugs categorized under the ATC group R03DX + D11AH05 (dupilumab), totaled 2280 cases, representing 0.38% of the entire FEDRA case database. These reports originate from various sources, primarily from the Marketing Authorization Holder (MAH), accounting for most notifications.

Regarding the notifier type, physicians contributed to the highest proportion of notifications at 48%, sourced from both Healthcare Surveillance Entities (SEFV-H) and the Pharmaceutical Industry. Users themselves, predominantly through the MAH, accounted for 34% of the cases. Given that these medications are exclusively prescribed in hospital settings, notifications from healthcare professionals, including physicians and pharmacists, primarily originate from such institutions. Unsurprisingly, most patients were adults aged between 18 and 65 years, with women comprising twice the number of cases compared to men.

Concerning the severity of reported ADRs, 36% (840 cases) were classified as serious by the notifier, health professional, or patient. Clinically relevant conditions accounted for 29% (669 cases) of these serious reports, including 23 fatalities, 65 life-threatening situations, 35 instances of persistent disability, and 239 hospital admissions or prolonged stays (Table 2).

When analyzing the evolution or clinical outcome of the reported reactions, significant disparities were noted in the available information between the notifications originating from Marketing Authorization Holders (MAHs) and those from Healthcare Surveillance Entities (SEFV-H), as illustrated in Table 3.

### 3.2. Active Ingredient

Table 4 presents the number of cases recorded in FEDRA for each active ingredient, accompanied by the percentages of serious adverse drug reactions (ADRs) and warning cases. Furthermore, the table displays the distribution of patients with ADRs by sex for each suspected drug. Importantly, the variation in the number of serious and warning cases among the active ingredients shown in Table 4 does not imply a superior or inferior safety profile; instead, it reflects differences in reporting frequency.

While these data do not directly derive from the present FEDRA case review, it was imperative to assess the safety data outlined in the SmPC for each drug. Hence, we conducted a comprehensive review of ADRs (Supplementary Table S1), emphasizing that all the ADRs documented in the SmPC have also been reported. Moreover, Table 5 delineates additional ADRs that have already been reported to FEDRA but are not presently documented in the SmPC, highlighting potential gaps in the currently available safety information.

#### 3.2.1. Omalizumab

Based on information from the Xolair^®^ Summary of Product Characteristics (SmPCs), the most frequent adverse drug reactions (ADRs) associated with omalizumab, occurring in up to 1 in 10 patients, include headaches and injection site reactions characterized by pain, swelling, redness, and itching. In children aged 6 to 12 years with allergic asthma, fever and upper abdominal pain were commonly reported. For patients with chronic urticaria, joint pain, sinusitis, and upper respiratory tract infections were predominant ADRs. Similar patterns were observed in patients with chronic rhinosinusitis with nasal polyps, where upper abdominal pain, dizziness, and joint pain were significant concerns.

Additional ADRs reported in FEDRA beyond those listed in the Xolair^®^ SmPC, include the following:

**Cutaneous Disorders:** Skin disorders were reported in 25% of the cases in FEDRA, totaling 166 cases out of 672. Notable additional ADRs reported include atopic dermatitis, purpura, and hyperhidrosis.

**Nervous System:** Beyond the known ADRs like headache, syncope, paresthesia, dizziness, and drowsiness, which collectively accounted for 18% of the notifications in FEDRA, the vascular disorders of the central nervous system (such as cerebrovascular accidents, transient ischemic attacks, and ischemic strokes), tremors, dyskinesias, movement disorders, and seizure disorders were reported.

**Musculoskeletal and Connective Tissue Disorders:** While arthralgia, myalgia, joint swelling, and systemic lupus erythematosus are recognized the ADRs of omalizumab, additional musculoskeletal ADRs reported in FEDRA included back pain, extremity pain, muscle weakness, muscle spasms, musculoskeletal stiffness, and discomfort in limbs.

**Neoplastic Disorders:** Although not described in the Xolair^®^ SmPC, FEDRA reported 67 cases (10% of the total) linking omalizumab to neoplastic disorders. Most reported were the malignant neoplasms of the breast, with other malignancies such as colorectal neoplasms, unspecified lymphomas, and respiratory tract and pleural neoplasms also noted.

**Immune System:** In FEDRA, 9% of the total cases reported adverse reactions related to the immune system, including optic neuritis, multiple sclerosis, and Sjögren’s syndrome, which are not listed in the Xolair^®^ SmPC.

**Complementary Investigations:** Although not detailed in the Xolair^®^ SmPC, FEDRA reported 45 cases involving disorders related to complementary examinations, with decreased weight being the notable term of interest.

**Infections and Infestations:** While infections such as pharyngitis and parasitic infections are considered uncommon or rare in the Xolair^®^ SmPC, FEDRA reported 43 cases (6% of the total) of the suspected ADRs related to infections, including notable instances of herpes zoster virus infections.

**Vascular Disorders:** The Xolair^®^ SmPC mentions rare vascular disorders like postural hypotension and flushing; however, FEDRA reported 34 cases (5% of the total) including deep vein thrombosis, which is not listed.

**Cardiac Disorders:** Although not referenced in the Xolair^®^ SmPC, omalizumab was associated with 29 cases (4% of the total) of the reported cardiac disorders in FEDRA, including increased heart rate and coronary ischemic events such as angina pectoris and myocardial infarction.

**Blood and Lymphatic System Disorders:** The Xolair^®^ SmPC mentions potential hematologic effects like idiopathic thrombocytopenia; FEDRA reported 25 cases (4% of the total) with suspected omalizumab-related blood disorders such as anemia and lymphadenopathy.

**Pregnancy, Puerperium, and Perinatal Conditions:** While the Xolair^®^ SmPC acknowledges the placental crossing of omalizumab without fetal toxicity risks, FEDRA reported 19 cases (3% of the total) including the instances of abortion.

**Eye Disorders:** Although not detailed in the Xolair^®^ SmPC, FEDRA reported 19 cases (3% of the total) of eye disorders, including edema of the eye and eyelid.

**Psychiatric Disorders:** No psychiatric disorders are listed in the Xolair^®^ SmPC; however, FEDRA reported 15 cases (2% of the total) linking omalizumab to psychiatric disorders, predominantly anxiety related.

**Reproductive System and Breast Disorders:** The Xolair^®^ SmPC does not include terms related to these systems; however, FEDRA reported 13 cases (2% of the total) involving disorders such as menstrual cycle irregularities.

**Metabolic and Nutritional Disorders:** These were not mentioned in the Xolair^®^ SmPC but accounted for nearly 2% of the cases in FEDRA, with decreased appetite being of notable concern.

**Ear and Labyrinth Disorders:** While not detailed in the Xolair^®^ SmPC, FEDRA reported eight cases (1% of the total) linking omalizumab to the disorders of the ear and labyrinth, notably including hearing loss.

#### 3.2.2. Mepolizumab

The most reported ADRs in the Nucala^®^ Summary of Product Characteristics (SmPCs) include headache, infections (lower respiratory tract infections, urinary tract infections, and pharyngitis), hypersensitivity reactions, nasal congestion, upper abdominal and back pain, eczema, injection site reactions, fever, and rare cases of anaphylaxis.

In the FEDRA database, 499 cases of adverse reactions linked to mepolizumab have been reported. These cases are categorized by frequency:

**Respiratory Issues:** Accounting for 30% of the cases (n = 166), this category includes known reactions as well as the exacerbations of treated conditions.

**Skin and Subcutaneous Reactions:** Also comprising 30% of the cases, these mostly align with hypersensitivity reactions described in the product information. Of particular interest are eight cases of alopecia.

**Neurological Symptoms:** Representing 26% of the cases, the majority include headaches and dizziness. Additional symptoms not specified in the product information are as follows:

▪ A total of 15 cases of paresthesia, hypoesthesia, or dysesthesia, with 5 cases deemed severe.
▪ A total of 6 cases of muscle weakness and atrophy, 3 of which were severe.
▪ A total of 5 cases of tremor, 3 of which were severe.
▪ In total, 4 cases of central nervous system (CNS) vascular disorders, all serious.

**Immune System Reactions:** Comprising 21% of the cases (n = 105), this category predominantly includes hypersensitivity reactions and anaphylaxis already described in the SmPC. However, there were five cases that may indicate a relationship with other diseases or autoimmune symptoms, including lichen planus, vasculitis, telangiectasia, pemphigoid, and diseases related to immunoglobulin G4.

**Musculoskeletal Symptoms:** There were 27 cases of arthralgia (5% of all the reported cases), with 7 cases classified as severe.

**Cardiac Issues:** These are not outlined in the Nucala^®^ SmPC. Nonspecific symptoms were noted in 55 cases, with 7 serious arrhythmias reported, which might be related to the underlying disease.

**Vascular Disorders:** This category included 67 cases (13%), coded with 30 ADR terms including vasculitis, thrombosis, purpura, cyanosis, telangiectasia, and hypertension, all of which are described in hyper-eosinophilic conditions. Of these, nine cases included terms such as deep vein thrombosis, stroke, and/or pulmonary embolism.

**Infections and Infestations:** Comprising 13% of the cases, this category included upper respiratory infections and one case of visceral leishmaniasis. Herpes zoster infection was notable for its statistical significance.

**Gastrointestinal Symptoms:** These are not mentioned in the Nucala^®^ SmPC. Among the 64 cases describing gastrointestinal symptoms, 21 were considered serious clinical conditions.

**Psychiatric Issues:** These were not covered in the Nucala^®^ SmPC. The 29 cases reported in FEDRA were insufficient to establish a clear ADR pattern.

**Neoplasms:** FEDRA reported 24 cases (5%) involving neoplasms, including 2 cases of male breast cancer, 3 cases of lung cancer, and 5 cases of gastrointestinal tract cancers, which were particularly noteworthy.

#### 3.2.3. Benralizumab

FEDRA contains 588 cases reporting adverse reactions from both spontaneous notification programs and observational studies where benralizumab was the suspected drug. Consistent with the benralizumab SmPC, headaches (including migraine) were the most frequently reported ADR, accounting for 14.6% (86 cases), followed by pharyngitis (including aphonia, tonsillitis, dysphagia, oropharyngeal pain, throat dryness, nasopharyngitis, and laryngeal irritation), with 16.15% (95 cases).

**General Disorders and Administration Site Conditions:** This was the most frequently reported category, comprising 42% (246 cases) of all the cases. Fatigue was mentioned in 55 cases, with 9 cases being classified as severe asthenia.

**Respiratory, Thoracic, and Mediastinal Disorders:** These accounted for 34% (200 cases) of the total cases. However, the symptoms described could be related to the underlying disease being treated, so they are not detailed.

**Neurological Disorders:** Symptoms were present in 26% of the cases (151 cases). However, most of these terms corresponded to headache (82 cases), a reaction already documented in the SmPC. The rest were too nonspecific and scattered for analysis.

**Skin and Subcutaneous Tissue Disorders:** These accounted for 16% of the cases (116 cases), with most falling under hypersensitivity reactions described in the product information. However, there were six cases of alopecia that may be of interest.

**Musculoskeletal and Connective Tissue Disorders:** They represented 15% of all the cases (86 cases), with myalgia being the most notable (29 cases, 14% of them severe), followed by arthralgia (18 cases, 22% of which were regarded as severe).

**Gastrointestinal Disorders:** These accounted for 14% of all the cases (85 cases), most corresponding to the disorders listed in the product information or being too scattered for analysis. However, there were 14 cases with terms related to abdominal pain: 7 of abdominal discomfort, 5 of abdominal pain (one severe), and 5 of upper abdominal discomfort.

**Immune System Disorders:** This category accounted for 13% of the cases (75 cases), primarily comprising hypersensitivity reactions (systemic allergic reaction and cutaneous manifestations) and anaphylaxis.

**Infections:** These represented 11% of the cases (68 cases). While many terms referred to pharyngeal infections already described in the SmPC, other infection-related terms appeared in 39 cases (more than half of all the infections), with 19 of them being severe. Notably, terms related to pneumonia were reported in five cases, including unspecified pneumonia, streptococcal pneumonia, fungal pneumonia, pneumonia due to COVID-19 (one case each), or other terms suggestive of pneumonia (pleural effusion, productive cough, etc.).

**Cardiac Disorders:** These accounted for 11% of all the notified cases (66 cases), with symptoms possibly related to coronary ischemia being notable: 7 cases of chest pain, 9 of chest discomfort, and 1 case of Kounis syndrome.

**Vascular Disorders:** The terminology dispersion in this category hinders interpretation, but blood pressure disorders stand out, with five cases of hypertension and five cases of hypotension.

**Psychiatric Disorders:** Although not described in the product information, there were 45 reported cases (8%), with sleep disorders being notable, accounting for 22 of the 52 terms (nearly half) grouped under psychiatric disorders. Other remarkable terms were those related to anxiety (four cases) and nervousness (four cases) or mood disorders (four cases).

**Other Reactions Not Described in the SmPC:** These included both weight increase (three cases) and decreased appetite (three cases).

#### 3.2.4. Dupilumab

In FEDRA, 537 cases were associated with the drug dupilumab. Upon reviewing the SmPC, the most frequent adverse reactions included injection site reactions (such as erythema, swelling, itching, pain, and swelling), conjunctivitis, allergic conjunctivitis, arthralgia, oral herpes, and eosinophilia. An additional adverse reaction of bruising at the injection site was reported in the US. Rare cases of serum sickness, serum sickness-like reactions, anaphylactic reactions, and ulcerative keratitis have also been reported.

**Skin and Subcutaneous Tissue Disorders:** These were the most frequent suspected ADRs, accounting for 29% (159 cases) of all the notified cases. The ADRs not described in the SmPC included alopecia/alopecia areata with 21 cases and psoriasis with 11 cases.

**Infections and Infestations:** The second most reported category, with 28% (153 cases) of the cases recorded in FEDRA. Although the dupilumab SmPC mentions infections such as conjunctivitis, oral herpes, and potential impacts on helminthic infections, FEDRA reported additional cases like pneumonia (11 cases), lower respiratory tract infections (3 cases), and cellulitis (3 cases).

**General Disorders and Conditions at the Local Administration Site:** FEDRA records 136 cases (25%) in this category. The dupilumab SmPC addresses these disorders, but FEDRA highlights the additional cases of pyrexia (14 cases), malaise (12 cases), asthenia (9 cases), and fatigue (9 cases).

**Ocular Disorders:** FEDRA reported 88 cases (16%) secondary to dupilumab, including symptoms like tearing, discomfort, and hyperemia, which may indicate the presence of the already described ADRs such as allergic conjunctivitis, keratitis, blepharitis, ocular pruritus, dry eye, and ulcerative keratitis listed in the SmPC.

**Nervous System Disorders:** Not covered in the dupilumab SmPC, FEDRA records 67 cases (12%), highlighting headaches (25 cases), dizziness (10 cases), and syncope (5 cases).

**Musculoskeletal and Connective Tissue Disorders:** FEDRA lists 58 cases (10%), with arthralgia being the most common, consistent with the SmPC. Other reported symptoms include myalgia (five cases), bone pain (five cases), and limb pain (five cases).

**Investigations:** FEDRA documents 47 cases (8%) describing abnormalities, including decreased weight (5 cases), which is not mentioned in the SmPC.

**Gastrointestinal Disorders:** Not listed as possible ADRs in the SmPC, FEDRA associates 35 cases (6%) with dupilumab, including vomiting and nausea (13 cases), diarrhea (5 cases), and Crohn’s disease (3 cases).

**Blood and Lymphatic System Disorders:** Described in the SmPC, FEDRA reports 35 cases (6%), with most involving eosinophilia (18 cases) already mentioned in the SmPC, and lymphadenopathy (11 cases) not previously described.

**Vascular Disorders:** FEDRA records 23 cases (4%), including erythema (7 cases) and vasculitis (5 cases), none of which were previously reported in the SmPC.

**Psychiatric Disorders:** FEDRA notes 21 cases (3%), with the most prevalent being anxiety, stress, and nervousness, totaling 11 cases together.

**Immune System Disorders:** FEDRA includes 16 cases (2%), with the most characteristic being eosinophilic granulomatosis with polyangiitis (EGPA) with 3 cases.

**Cardiac Disorders:** FEDRA reports 11 cases (2%), with tachycardia (5 cases) being the most frequent.

**Metabolic and Nutritional Disorders:** Described in six cases (1%), with abnormal weight loss (two cases) and decreased appetite (two cases), notable and absent in the SmPC.

#### 3.2.5. Reslizumab

As the sole biologic drug in our group designated for intravenous administration, its usage is notably lower compared to other alternatives, even in the absence of consumption data. Currently, there have been only 30 suspected ADRs reported with this biologic drug, among which 19 were classified as serious. These cases encompassed several instances of arthralgia, some of which were clinically severe, along with seven reports of neoplasms, including two cases of glioma, and the occurrences of two thrombosis or pulmonary embolism.

#### 3.2.6. Tezepelumab

With its recent market introduction in September 2023, up to April 1, 2024, there have been only eleven cases of suspected ADRs associated with this biologic drug. Among them, five were deemed serious, with two requiring hospital admissions and three exhibiting clinically significant symptoms. The reported reactions align with those described in the Tezspire^®^ SmPc, encompassing arthralgia, myalgia, bronchospasm, cough, dyspnea, pruritus, erythema, and wheezing.

Additionally, Table 6 displays the values of the mentioned reporting disproportion of drug–ADR associations reported to FEDRA and not contained in the SmPC.

## 4. Discussion

This investigation is the first to evaluate the real-life safety profile of approved asthma biologics using the FEDRA database in Spain. Given the substantial variations in asthma phenotypes [12,48,49] and the heterogeneity in the use of asthma biologics across different regions and populations [50,51], obtaining an overview of the locally prevalent ADRs not currently addressed in the biological therapies’ SmPC is crucial.

Industry notifications are pivotal in the FEDRA database, comprising nearly 90% of the reported cases concerning these biologics. Approximately 40% of these notifications stem from industry-sponsored studies. Notably, SEFV-H excludes these study-based reports from its statistical analysis to prevent bias, thus potentially omitting a significant portion of unexpected adverse effects crucial for signal detection. Moreover, there are distinct differences in the quality and completeness of information between reports from MAH and SEFV-H. For instance, industry reports classify outcomes as unknown in over 40% of the cases, whereas SEFV-H accounts for less than 20% under similar circumstances. This discrepancy extends to critical data points such as patient demographics (age and sex), treatment duration, and the progression of the reported ADRs. Consequently, data sourced from industry notifications may provide less conclusive insights compared to those gathered directly by health system pharmacovigilance programs.

### 4.1. SmPC and Risk

The review of reports within the FEDRA database has also identified numerous risks not included in the SmPCs of these biologics. Additionally, ADRs documented in the SmPCs were often reported in significant quantities. There is a notable disparity between the limited safety information provided in the SmPCs and the comprehensive data yielded by the pharmacovigilance system. Moreover, the inconsistency in the ADR content among the various SmPCs is striking. For example, both mepolizumab and omalizumab include myalgia and arthralgia in their ADR profiles. In contrast, reslizumab lists only myalgia, while tezepelumab and dupilumab exclusively mention arthralgia. Surprisingly, benralizumab’s SmPC does not include musculoskeletal adverse effects, yet both myalgia and arthralgia have been reported with statistical significance in FEDRA, with many cases being serious (Supplementary Table S2).

Although pyrexia is mentioned in the SmPCs of omalizumab, mepolizumab, and benralizumab, it is absent in dupilumab’s SmPC, yet it has been equally reported across these medications. Alopecia is described in the omalizumab SmPC but not for the other biologics, although it has been reported with all of them, even in a statistically significant manner with mepolizumab, benralizumab, and dupilumab [52,53]. The mechanism of action of dupilumab may contribute to sebaceous gland atrophy and subsequent alopecia, which might also apply to the other drugs studied [54,55]. The infections of the lower respiratory tract are solely listed in mepolizumab’s SmPC, despite being widely reported with omalizumab, benralizumab, and dupilumab. These discrepancies highlight the importance of comprehensive reporting and analysis within pharmacovigilance systems. Although it is a striking finding, we do not expect identical information in each document nor suggest a class effect as each medication operates through distinct mechanisms, leading to differences in their adverse event profiles [56,57]. Understanding these distinctions may enhance the comprehension of medication safety profiles and inform clinical decision making.

### 4.2. ADRs and Underlying Disease

The clinical significance of ADRs reported with these biologics indicates that some symptoms occur with each active ingredient in this group. This finding prompts us to consider whether the symptoms may be related to the underlying disease, any biological therapy, or if they are attributable to each drug. For instance, fatigue and asthenia, frequently reported with these drugs, may be related to the underlying disease. Similarly, the avoidance of discussing respiratory-related symptoms underlines the importance of considering any ADR likely to occur in patients with severe uncontrolled asthma.

### 4.3. ADRs and Eosinophilic Granulomatosis with Polyangiitis (EGPA)

Reports concerning immune system disorders in FEDRA included 3 cases of EGPA with dupilumab, 3 with benralizumab, 2 with mepolizumab, and 11 with omalizumab. The SmPC of dupilumab mentions EGPA cases in both drug-treated and placebo-treated patients, suggesting a possible relationship with corticosteroid reduction, which could serve as an alternative explanation applicable to all the drugs. However, this implies that unless the clinician confirms the discontinuation of corticosteroid use in a patient, the case of EGPA may be considered potentially linked to the drug under scrutiny. In addition, the disruption produced by dupilumab may induce excessive eosinophilic inflammation, contributing to the development of EGPA [54]. To further complicate matters, certain biological drugs not only may have the potential to trigger EGPA but can also serve as treatments for the condition [58,59,60,61]. Although causality has not been established, caution is recommended when prescribing dupilumab for uncontrolled asthma with characteristics that may indicate EGPA [54,62].

### 4.4. Neoplastic Disorders and Biological Therapy

The potential for tumor development when using a drug that impacts the immune response is concerning. The SmPC of omalizumab states that “No formal carcinogenicity studies have been performed with omalizumab”, and neither benign nor malignant neoplasms are described. However, some controversies have been raised as its long-term use has recently been linked to potential cancer risk [63,64,65,66]. FEDRA registered 67 cases linking omalizumab with neoplastic disorders, representing 10% of its total cases. The evidence from the literature is inconclusive [67]. A similar situation occurred with mepolizumab, where neoplasms reported to FEDRA accounted for 24 cases, including male breast cancer (two cases), lung (three cases), and gastrointestinal tract cancers (five cases). With benralizumab and dupilumab, different types of neoplasms were reported but without any available reference in the SmPC. For benralizumab, there were 11 cases, and for dupilumab, 10 cases, both accounting for 2% of the total cases.

### 4.5. ADRs and Vascular Disorders

Central nervous system vascular disorders, including cerebrovascular accidents, transient ischemic attacks, and ischemic strokes, have been reported with both omalizumab and mepolizumab. These events, included in the product information for the US by the FDA several years ago, were not incorporated in the SmPC for either drug [68,69]. Cases of deep vein thrombosis have also been reported with both omalizumab and mepolizumab. Additionally, pulmonary embolism has been described with mepolizumab and occasionally with reslizumab [70]. Omalizumab has been associated with cardiac disorders, notably the ischemic alterations of coronary arteries. Similarly, there are cases of ischemic coronary disorders with benralizumab; however, this information was not included in the SmPC for either drug. Some cases of vascular disorders associated with mepolizumab exhibited terms including vasculitis, thrombosis, purpura, cyanosis, and telangiectasia, which are also described in hyper-eosinophilic conditions, suggesting another instance of overlap with the underlying condition [71,72].

### 4.6. Specific Comments on ADRS for Active Ingredients in Biological Agents Primarily Indicated for Severe Refractory Asthma

#### 4.6.1. Omalizumab

Safety issues with omalizumab accumulate the most cases, possibly due to its status as the oldest drug among those reviewed. In addition to the vascular conditions already described, the association with deafness [73] and various autoimmune conditions (multiple sclerosis, Sjögren’s syndrome, and optic neuritis) stands out, requiring more specific studies due to their clinical relevance. The association of spontaneous abortion with omalizumab also exhibits a statistical association, demanding further investigation, a situation not observed with the rest of the biologics for severe asthma [74].

#### 4.6.2. Mepolizumab

Among the cases reported with mepolizumab, the association of paresthesias, dysesthesias, and hypoesthesias stands out, as these are not described with the other biologics in this group. The occurrence of herpes zoster (six cases) holds statistical significance [67,75,76,77], and PRAC has included this ADR in the side effects section of the mepolizumab SmPC as of April 2024.

#### 4.6.3. Benralizumab

Safety concerns regarding benralizumab appear underestimated. The SmPC provides scant information: the ADR table includes only seven terms grouped into four SOCs. However, ADR reporting associated with this drug presents a different narrative in this study, something that also has been reported by former research [78,79]. It is particularly striking that infections, a significant concern for a drug affecting the immune system, accounted for 11% of the reported cases. Conversely, the benralizumab SmPC describes only pharyngitis, while other types of infections have been previously overlooked [75,80,81] despite some studies addressing them [82,83]. Another type of reaction that should be noted are sleep disorders due to benralizumab, a condition that may be easily assessed during the clinical interview with a simple question. However, it should be noted that all these findings may not align with the conclusions drawn in the other scientific literature [84].

#### 4.6.4. Dupilumab

The sex distribution of the reported suspected ADRs for these drugs generally mirrors that of patients with SA, with a higher frequency observed in females (3:1). However, dupilumab shows a different ratio (1:1), likely due to its initial indication for uncontrolled severe atopic dermatitis, which may exhibit a distinct sex distribution [85]. The elevated reporting of conjunctivitis and eye symptoms with dupilumab could be linked to its initial indication for atopic dermatitis [38]. Although conjunctivitis is not typically associated with asthma but rather with atopic eczema, studies have noted its occurrence in patients treated with dupilumab compared to placebo or other immunosuppressants [86,87,88]. Similar overlaps may occur with skin and subcutaneous tissue disorders, which were the most frequently reported ADRs with dupilumab. However, the reporting of alopecia/alopecia areata (21 cases), not mentioned in the SmPC, may not be directly attributable to underlying atopic eczema [53,89,90], suggesting a different causative mechanism from dermatitis. Metabolic and nutritional disorders were reported in six cases (1%) with dupilumab, including the notable occurrences of abnormal weight loss (two cases) and decreased appetite (two cases), which are absent from the SmPC. Interestingly, weight gain, previously described but not included in the SmPC, has also been noted [91]

## 5. Limitations

Studies relying on the spontaneous reporting of ADRs face several significant limitations, notably underreporting to the pharmacovigilance system, which leads to an underestimation of the true frequency of drug-related adverse events. Reporting rates can fluctuate due to factors such as the type and severity of the reaction, the specific medications involved, and whether the drug is newly marketed or established [42,92,93]. Additionally, it is important to note that the cases analyzed in this study include patients treated with these drugs regardless of their underlying condition. While this may be perceived as a limitation, several considerations support the validity of our approach. Our primary aim was to comprehensively assess the safety profiles of these approved drugs, minimizing bias related to underlying conditions. The documentation of biologic drug indications is often incomplete, and studies focusing solely on indications may overlook a significant number of reports, potentially up to 70% in some cases [94]. However, our study did not identify any new ADRs that were not already described in our investigation, supporting the completeness of our data. These findings underline the importance of routinely including indication data in pharmacovigilance reporting.

## 6. Conclusions and Future Research Directions

The clinical significance of ADRs is influenced by various factors, including the number of reported cases. ADRs can be defined by single or multiple terms, making it challenging to establish a clear threshold for clinical significance. However, many ADRs discussed in the study show statistical significance through disproportionality analysis, indicating their clinical relevance despite limitations in including cases from industry studies in such analyses. Pharmacovigilance activity, based on spontaneous reporting programs for suspected ADR, serves as an effective system for gaining accurate and early insights into the profile of ADR in real-world settings. The generation and validation of signals in pharmacovigilance enable the generation of new safety hypotheses and contribute to the ongoing safety surveillance of drugs post-marketing. Real-world studies offer a valuable tool for the post-marketing monitoring of biological drugs, enhancing our understanding of their effectiveness and safety in everyday clinical practice, thereby bolstering clinicians’ confidence among clinicians in their utilization of biologics in SA.

Despite the biologics’ overall safety, this review of the Spanish Pharmacovigilance database reveals numerous serious cases not documented in the SmPC, warranting detailed investigation as pharmacovigilance signals. Addressing these associations is a commitment for our research team in the future. Furthermore, for future reviews, exploring adverse effects following the prolonged use of these biological medications, particularly with usage periods exceeding 2 years post-marketing, appears worthwhile.

Considering the relevance of enhancing drug safety knowledge, it is imperative to encourage the active participation and engagement of patients, healthcare professionals, pharmaceutical companies, and regulatory bodies in reporting suspected ADRs through the promotion of side effect-reporting programs. The collaboration between CAFV and clinical areas like the Allergy Department at our Institution has proven instrumental in achieving clinically relevant results, emphasizing the significance of such partnership in advancing drug safety knowledge.

## Figures and Tables

**Table 1:** Biological drugs under study.

Biological Drugs (Date of Approval)	ATC-GTER Classification	Approved Indications	Brand Name
Omalizumab (25 October 2005)	R03DX05—other systemic drugs for obstructive airway diseases	Allergic asthma convincingly mediated by IgE Chronic spontaneous urticaria Chronic rhinosinusitis with nasal polyps	Xolair^®^ (Novartis International AG, Basel, Switzerland)
Mepolizumab (2 December 2015)	R03DX09	Severe refractory eosinophilic asthma Chronic rhinosinusitis with nasal polyps Eosinophilic granulomatosis with polyangiitis Hyper-eosinophilic syndrome	Nucala^®^ (GlaxoSmithKline plc, Brentford, UK)
Reslizumab (15 May 2016)	R03DX08	Insufficiently controlled severe eosinophilic asthma	Cinqaero^®^ ▼ (Teva Pharmaceutical Industries, Petah Tikva, Israel)
Benralizumab (12 February 2018)	R03DX10	Severe eosinophilic asthma	Fasenra^®^ (AstraZeneca PLC, Cambridge, UK)
Tezepelumab (19 September 2022)	R03DX11	Severe asthma	Tezspire^®^ ▼ (Amgen Inc., Thousand Oaks, CA, USA and AstraZeneca PLC, Cambridge, UK)
Dupilumab (26 September 2017)	D11AH—agents for dermatitis, excluding corticosteroids	Moderate to severe atopic dermatitis Severe uncontrolled eosinophilic asthma Chronic rhinosinusitis with nasal polyps Prurigo nodularis Eosinophilic esophagitis	Dupixent^®^ (Sanofi, Paris, France, and Regeneron Pharmaceuticals, Inc., Tarrytown, NY, USA)

**▼** medicines under additional monitoring in the European Union.

**Table 2:** Origin of cases and seriousness. Each case may have multiple origins. Combining the cases of MAH origin with those of SEFV origin will result in a total number of cases that exceeds the actual number.

Origin of Cases (n)	Non-Serious n (%)	Serious * n (%)
SEFV-H (361)	154 (43%)	207 (57%)
MAH (1985)	1506 (76%)	479 (24%)

* Seriousness criteria: A case is considered serious if it meets at least one of the following: it causes death (fatal); it endangers the patient’s life; it requires admission or prolonged hospital stay; it results in disability or persistent disability; it causes a congenital anomaly or defect; or it has not caused any of the above but is considered clinically or medically significant. SEFV-H: Spanish System of Human Use Medicines Pharmacovigilance; MAHs: Marketing Authorization Holders.

**Table 3:** Outcome of adverse drug reactions according to the origin of data.

Origin of Cases (n)	Recovered	In Recovery	Recovered with after-Effects	Not Recovered	Mortal	Unknown
SEFV-H (361)	40%	25%	2%	16%	1%	17%
MAH (1985)	25%	8%	0.40%	22%	1%	43%

SEFV-H: Spanish System of Human Use Medicines Pharmacovigilance; MAHs: Marketing Authorization Holders.

**Table 4:** Number of the reported cases and seriousness for each active ingredient. Each case may have multiple origins. Combining the cases of MAH origin with those of SEFV origin will result in a total number of cases that exceeds the actual number.

Biological Drug	FEDRA (n)	Serious * Cases	Males	Females	Warning ** Cases
Omalizumab	672	61%	27%	65%	10.1%
Mepolizumab	497	32%	24%	66%	3.6%
Reslizumab	35	54%	17%	74%	17%
Benralizumab	588	18%	23%	75%	2.2%
Tezepelumab	3	33%	33%	66%	-
Dupilumab	536	29%	47%	45%	5,6%

* Seriousness criteria: a case is considered serious if it meets at least one of the following: it causes death (fatal); it endangers the patient’s life; it requires admission or prolonged hospital stay; it results in disability or persistent disability; it causes a congenital anomaly or defect; or it has not caused any of the above but is considered clinically or medically significant. ** warning cases: cases that involve unknown adverse reactions (not included in the SmPC) and that, due to their severity, are likely to generate a pharmacovigilance signal.

**Table 5:** Reported ADRs not collected in the SmPC. Bold characters reflect disproportionally reported data, indicating statistically significant associations. The number of cases (in brackets) for each diagnosis is not standardized and varies based on the total reports and reporter preferences. Comparisons between the drugs are not valid. Tepezelumab data are excluded due to limited cases currently available in FEDRA.

	Omalizumab	Mepolizumab	Reslizumab	Benralizumab	Dupilumab
Symptom Grouping	Diagnosis (No. of Cases)	Diagnosis (No. of Cases)	Diagnosis (No. of Cases)	Diagnosis (No. of cases)	Diagnosis (No. of Cases)
Blood and lymphatic system disorders	Anemic disorders (7) Lymphadenopathy (4)				Lymphadenopathy (11)
Cardiac disorders	**Ischemic coronary artery disorders** (six cases, including acute myocardial infarction) **Pericarditis** (4)			Ischemic coronary artery disorders (17)	
Ear and labyrinth disorders	**Deafness** (four, two of them specified as “Deafness neurosensory”)				
Eye disorders	Eyelid edema (4)				
Gastrointestinal disorders	Abdominal pain or discomfort (11)			Abdominal pain or discomfort (14)	Vomiting and nausea (13) Diarrhea (5) **Crohn’s disease** (3)
General disorders and administration site conditions		Fatigue–asthenic conditions (43)		Fatigue–asthenic conditions (55)	Fatigue–asthenic conditions (18) Pyrexia (14) Malaise (12)
Immune system disorders	**Multiple sclerosis** (3) **Sjögren’s syndrome** (3)				Eosinophilic granulomatosis with polyangiitis (EGPA) (3)
Infections and infestations	**Herpes viral infect**. (8) Lung and lower resp. tract infection (10)	Herpes viral infect * (7)		Infective pneumonia (9)	**Infective pneumonia** (7) Cellulitis (3)
Metabolism and nutrition disorders	Decreased appetite (3) Decreased weight (4)				Decreased appetite (2) Decreased weight (2)
Musculoskeletal and connective tissue disorders	Back pain (9) **Muscular weakness** (4) **Muscle spasms** (6) **Musculoskeletal stiffness** (5)	Arthralgia * (27)	**Arthralgia** (11)	Arthralgia (29) Myalgia (18) Muscle spasms (3)	Myalgia (5) **Bone pain** (4) Limb pain (5)
Neoplasms benign, malignant, and unspecified (incl cysts and polyps)	**Breast and nipple neoplasm malignant** (16) **Lymphomas unspecified** (4) **Colorectal neoplasm malignant** (7) **Respiratory tract neoplasm malignant cell type unspecified**. (7)	**Breast and nipple neoplasm malignant** (3, male breast cancer 2) **Malignant neo. GI NEC** (4) **Respiratory tract neoplasm malignant cell type unspecified** (3)	Glial tumors malignant (2)	Malignant neo.GI NEC (3) Respiratory tract neoplasm malignant cell type unspecified (2)	
Nervous system disorders	**Cerebrovascular accident** (8) **Gait disturbance** (3) **Gait inability** (1) **Movement disturbances** (eleven cases, including three cases of hypokinesia and five of tremor) **Optic neuritis** (3)	Cerebrovascular accident (3) Paraesthesias (8) Dysaesthesias (4) Hypoaesthesias (4) Tremor (5)			Headaches (25) Dizziness (10) Syncope (5)
Pregnancy, puerperium, and perinatal conditions	**Abortion spontaneous and gestational fetal death** (8)				
Psychiatric disorders	Anxiety, stress, and nervousness (4)			Sleep disorders (21)	Anxiety, stress, and nervousness (11)
Reproductive system and breast disorders	Menstrual cycle disorders (5)				
Respiratory, thoracic, and mediastinal disorders	Pulmonary embolism and thrombosis (5)	Pulmonary embolism and thrombosis (1)	Pulmonary embolism and thrombosis (2)		Eosinophilic pneumonia (1)
Skin and subcutaneous tissue disorders	**Atopic dermatitis** (4) Purpura (3) Hyperhidrosis (3)	**Alopecia** (8)	Alopecia (1)	**Alopecia** (4)	**Alopecia** (15)
Vascular disorders	**Deep vein thrombosis** (5)	Deep vein thrombosis (3)		Hypertension (5) Hypotension (5)	Vasculitis (5)

* The EMA Pharmacovigilance Committee (PRAC) in April 2024 has already included these adverse drug reactions in the side effects section of the summary of product characteristics for mepolizumab.

**Table 6:** Value of the disproportion of reporting for each of the drug–ADR associations signaled as statistically significant.

Association	N	Lower Limit ROR *	Lower Limit CI ^	X2 ^†^
Omalizumab–Acute myocardial infarction (PT: acute myocardial infarction, acute coronary syndrome, and Kounis’ syndrome)	6	1.48	0.23	9.25
Omalizumab–Angina pectoris (PT)	4	2.97	0.45	23.05
Omalizumab–Pericarditis (PT: pericarditis; pleuropericarditis)	4	2.86	0.43	21.95
Omalizumab–Deafness (PT: deafness; neurosensorial deafness)	4	3.69	0.54	30.19
Omalizumab–Multiple sclerosis (PT)	3	2.75	0.13	18.45
Omalizumab–Sjögren’s syndrome (PT)	3	15.15	0.42	122.99
Omalizumab–Optic neuritis (PT)	3	3.28	0.19	22.95
Omalizumab–Herpes viral infection (PT: oral herpes, herpes zoster, eczema herpeticum, and herpes virus infections.)	8	1.10	0.00	5.7
Omalizumab–Muscular weakness; back pain; muscle spasms; joint stiffness (PTs)	18	1.11	0.09	5.85
Omalizumab–Breast and nipple neoplasm malignant (PTs: hormone receptor-positive breast cancer, breast cancer, invasive lobular breast carcinoma, invasive breast carcinoma, and invasive ductal breast cancer)	16	23.33	2.84	540.78
Omalizumab–Colon cancer (PT)	7	12.49	1.62	162.52
Omalizumab–Lymphoma (PT)	4	1.64	0.10	9.78
Omalizumab–Lung adenocarcinoma and lung malignant neoplasm (PTs)	7	8.27	1.48	104.16
Omalizumab–Cerebrovascular accident, ischemic stroke, and transient ischemic attack (PTs)	8	2.48	0.82	24.74
Omalizumab–Gait disturbance, gait inability, mobility decreased, and hypokinesia (PTs)	8	1.44	0.29	9.54
Omalizumab–Spontaneous abortion and gestational fetal death (PTs)	8	4.05	1.21	48.52
Omalizumab–Atopic dermatitis (PT)	4	8.06	0.77	73.97
Omalizumab–Deep vein thrombosis (PT)	5	1.47	0.14	8.71
Omalizumab–Embolic and thrombotic events (SMQ)	25	1.51	0.50	16.69
Mepolizumab–Arthralgia (PT) **	27	2.22	0.95	39.15
Mepolizumab–Herpes zoster (PT) **	6	2.42	0.63	20.81
Mepolizumab–Breast and nipple neoplasm malignant (HLT) and Breast malignant tumors (SMQ)	3	5.36	0.32	40.85
Mepolizumab–Gastrointestinal neoplasms malignant and unspecified (HLGT)	4	5.41	0.67	47.55
Mepolizumab–Respiratory and mediastinal neoplasms malignant and unspecified (HLGT)	3	5.98	0.34	46.21
Mepolizumab–Malignancies (SMQ)	16	2.86	1.19	44.32
Mepolizumab–Alopecia (PT)	8	3.88	1.17	45.66
Reslizumab–Arthralgia (PT)	10	6.28	1.42	77.96
Benralizumab–Alopecia (PT)	4	2.68	0.38	20.05
Dupilumab–Crohn’s disease (PT)	3	3.67	0.22	26.23
Dupilumab–Infective pneumonia (SMQ) [excluded COVID-19]	7	1.92	0.52	15.47
Dupilumab–Bone pain (PT)	4	2.02	0.23	13.52
Dupilumab–Alopecia (PT)	15	5.00	1.76	92.92

* The Reporting Odds Ratio (ROR) must be above 1 to have statistical significance. ^ The Information Component (CI) must be above 0 to have statistical significance. **^†^** The Chi-Square (X2) has no interval limits and gives information about the strength of the association (the higher the value, the higher the strength). Above 4, it is considered to be statistically significant, sustaining the association. ** The EMA Pharmacovigilance Committee (PRAC) of April 2024 has already included this ADR in the side effects section of the FT of Mepolizumab. SMQ: Standardized MedDRA Queries. PTs: Preferred Terms. HLGT: High-Level Group Term. HLT: High-Level Term.

## Data Availability

The data that support the findings of this study are available from Servicio Canario de la Salud; however, restrictions apply to the availability of these data, which were used under license for the current study, and so are not publicly available. The data are, however, available from the authors upon reasonable request and with the permission of Servicio Canario de la Salud.

## Supplementary Materials

The following supporting information can be downloaded at: https://www.mdpi.com/article/10.3390/jcm13144192/s1, Table S1. Adverse drug reactions currently described in the summary of product characteristics. The number of cases (in brackets) for each diagnosis is not standardized and cannot be compared between drugs as it largely depends on the total number of reports and reporter preferences. For tezepelumab and reslizumab, the data were limited, so ADRs previously described in the summary of product characteristics may not appear. Table S2. Variations regarding arthralgia and myalgia in the summary of product characteristics.
