# Supplementary material for: Real-World Safety Profile of Biologic Drugs for Severe Uncontrolled Asthma: A Descriptive Analysis from the Spanish Pharmacovigilance Database
Source: J Clin Med. 2024 Jul 18;13(14):4192. doi: 10.3390/jcm13144192 (PMC11277876; doi:10.3390/jcm13144192)
Supplement: Supplementary file 1 [file jcm-13-04192-s001.zip › jcm-3000828-supplementary.pdf]

Journal of
Clinical Medicine

MDPI

Supplementary Materials:

Table S1. Adverse drug reactions currently described in the Summary of Product Characteristics. The number of cases (in brackets) for each diagnosis is not standardized and cannot be compared between drugs as it largely depends on the total number of reports and reporter preferences. For tezepelumab and reslizumab, the data were limited, so ADRs previously described in the Summary of Product Characteristics may not appear.

	**Omalizumab**	**Mepolizumab**	**Reslizumab**	**Benralizumab**	**Tezepelumab**	**Dupilumab**
**Symptom Grouping**	Diagnosis (No. of cases)	Diagnosis (No. of cases)	Diagnosis (No. of cases)	Diagnosis (No. of cases)	Diagnosis (No. of cases)	Diagnosis (No. of cases)
Blood and lymphatic system disorders	Idiopathic thrombocytopenia (7 cases of thrombocytopenia without primary origin)					Eosinophilia (18)
Eye disorders						Allergic conjunctivitis. (13) Keratitis (11) Blepharitis (11) Eye pruritus (8) Dry eye (15) Ulcerative keratitis (3)
Gastrointestinal disorders	Upper abdominal pain (-)	Upper abdominal pain (7)				
General disorders and administration site conditions	Pyrexia (40) Injection site reactions. (20) Influenza-like illness (7) Swelling arms (-) Weight increase (9)	Pyrexia (35) Injection site reactions (39) Systemic non allergic administration- related reactions (43)		Pyrexia (51) Injection site reactions (50)	Injection site reactions (-)	Injection site reactions. (27)

	Omalizumab	Mepolizumab	Reslizumab	Benralizumab	Tezepelumab	Dupilumab
**Symptom Grouping**	Diagnosis (No. of cases)	Diagnosis (No. of cases)	Diagnosis (No. of cases)	Diagnosis (No. of cases)	Diagnosis (No. of cases)	Diagnosis (No. of cases)
	Fatigue-Asthenic conditions. (44)					
Immune system disorders	Anaphylactic reaction and other serious allergic conditions. (45) Anti-omalizumab antibody development (-) Serum sickness. (4)	Anaphylactic reaction and other serious allergic conditions (6)	Anaphylactic reaction (-)	Anaphylactic reaction (2) Hypersensitivity reactions (79)	Anaphylactic reaction and other serious allergic conditions (-)	Anaphylactic reaction (1) Angioedema (7) Serum sickness (1)
Infections and infestations	Pharyngitis (12) Parasitic infection (-)	Pharyngitis (24) Lower respiratory tract infection (16) Urinary tract infections (3)		Pharyngitis (95)	Pharyngitis (-)	Conjunctivitis (87) Oral herpes (4)
Investigations			Blood creatine phosphokinase increased (-)			
Musculoskeletal and connective tissue disorders	Myalgia (33) Arthralgia (59) Joint swelling (5) Systemic lupus erythematosus (3)	Myalgia (19) Back pain (24)	Myalgia (4)		Arthralgia (-)	Arthralgia (34)
Nervous system disorders	Headache (39) Syncope (-)	Headache (66)		Headache (82)		

	Omalizumab	Mepolizumab	Reslizumab	Benralizumab	Tezepelumab	Dupilumab
**Symptom Grouping**	Diagnosis (No. of cases)	Diagnosis (No. of cases)	Diagnosis (No. of cases)	Diagnosis (No. of cases)	Diagnosis (No. of cases)	Diagnosis (No. of cases)
	Paresthesia (14)<br>Somnolence (7)<br>Dizziness (21)					
Respiratory, thoracic, and mediastinal disorders	Allergic bronchospasm (17)<br>Coughing (19)<br>Laryngoedema (6)<br>Allergic granulomatous vasculitis (-)	Nasal congestion (8)				
Skin and subcutaneous tissue disorders	Photosensitivity (1)<br>Urticaria (68)<br>Rash (29)<br>Pruritus (34)<br>Alopecia (14)	Eczema (7)<br>Rash (19)			Rash (-)	Rash (22)
Vascular disorders	Flushing (4)<br>Postural hypotension (9 cases oh hypotension without mention of etiology)	Flushing (1)				

Table S2. Variations regarding arthralgia and myalgia in the summary of product characteristics.

Biological Drug	Present in SmPC		Present in reports	
Omalizumab	Arthralgia	Myalgia	Arthralgia	Myalgia
Mepolizumab	Arthralgia	Myalgia	Arthralgia	Myalgia
Dupilumab	Arthralgia		Arthralgia	Myalgia
Tezepelumab	Arthralgia		Arthralgia	Myalgia
Reslizumab		Myalgia	Arthralgia	Myalgia
Benralizumab			Arthralgia	Myalgia

Disclaimer/Publisher's Note: The statements, opinions and data contained in all publications are solely those of the individual author(s) and contributor(s) and not of MDPI and/or the editor(s). MDPI and/or the editor(s) disclaim responsibility for any injury to people or property resulting from any ideas, methods, instructions or products referred to in the content.
